# Vaginal *Candida albicans* infections: host–pathogen–microbiome interactions

**DOI:** 10.1093/femsre/fuaf013

**Published:** 2025-05-10

**Authors:** Marisa Valentine, Duncan Wilson, Mark S Gresnigt, Bernhard Hube

**Affiliations:** Department of Microbial Pathogenicity Mechanisms, Leibniz Institute for Natural Product Research and Infection Biology, Hans Knöll Institute, 23 Adolf-Reichwein-Straße, 07745, Jena, Germany; Medical Research Council Centre for Medical Mycology at the University of Exeter, University of Exeter, Geoffrey Pope Building Stocker Road, Exeter EX4 4QD, United Kingdom; Junior Research Group Adaptive Pathogenicity Strategies, Leibniz Institute for Natural Product Research and Infection Biology, Hans Knöll Institute, 23 Adolf-Reichwein-Straße, 07745, Jena, Germany; Department of Microbial Pathogenicity Mechanisms, Leibniz Institute for Natural Product Research and Infection Biology, Hans Knöll Institute, 23 Adolf-Reichwein-Straße, 07745, Jena, Germany; Institute of Microbiology, Friedrich Schiller University, 25 Neugasse, 07743, Jena, Germany; Cluster of Excellence Balance of the Microverse, Friedrich Schiller University Jena, 1 Fürstengraben, 07743, Jena, Germany

**Keywords:** *Candida albicans*, vulvovaginal candidiasis, immunopathology, lactobacilli, treatment strategies, infection models

## Abstract

*Candida albicans* is a fungus that colonizes the gut, oral, and vaginal mucosae of most humans without causing disease. However, under certain predisposing conditions this fungus can cause disease. *Candida albicans* has several factors and attributes that facilitate its commensal and pathogenic lifestyles including the transition from a yeast to a hyphal morphology, which is accompanied by the expression of virulence factors. These factors are central in candidiasis that can range from invasive to superficial. This review focuses on one example of a superficial disease, i.e. vulvovaginal candidiasis (VVC) that affects ~75% of women at least once with some experiencing four or more symptomatic infections per year (RVVC). During VVC, fungal factors trigger inflammation, which is maintained by a dysregulated innate immune response. This in turn leads to immunopathology and symptoms. Another unique characteristic of the vaginal niche, is its *Lactobacillus*-dominated microbiota with low species diversity that is believed to antagonize *C. albicans* pathogenicity. The importance of the interactions between *C. albicans*, the host, and vaginal microbiota during commensalism and (R)VVC is discussed in this review, which also addresses the application of this knowledge to identify novel treatment strategies and to study vaginal *C. albicans* infections.

## Teaser

Review of the interaction between *Candida albicans*, the host, and microbiota during vaginal infections, as well as vaginal infection models and treatment options

## The fungus *Candida albicans*

Approximately 5 million fungal species exist that can be associated with various environmental and host niches including those of water, soil, plants, animals, and humans (Blackwell 2011). Although the vast majority of fungi is nonpathogenic, certain fungal species can cause disease in humans (Blackwell 2011, Bongomin et al. 2017). Several species belonging to the *Candida* genus can cause human diseases, which was recently highlighted by the World Health Organization (WHO; WHO 2022). The WHO report ranked and categorized fungal pathogens based on several criteria including mortality rates, incidence, antifungal resistance, and treatment difficulties. *Candida albicans* was one of four fungal species categorized as critical priority.

Normally, *C. albicans* is a commensal that lives asymptomatically on mucosal surfaces, including those of the gut, mouth, and vagina, of most healthy humans (Ghannoum et al. 2010, Drell et al. 2013, Nash et al. 2017, Delavy et al. 2023). This yeast can be transferred from mother to child during birth resulting in it colonizing the human body from early on in life (Bliss et al. 2008). Depending on the presence of predisposing conditions, *C. albicans* colonizing the human body can become pathogenic and cause a variety of diseases ranging from severe, systemic, and life-threatening invasive candidiasis to mucosal diseases such as oropharyngeal candidiasis (OPC) in immunocompromised individuals and vulvovaginal candidiasis (VVC) in women without a compromised immune status (Papon et al. 2013, d’Enfert et al. 2021).

VVC is a disease of the vulval and vaginal mucosa caused by *Candida* species, predominantly *C. albicans*, that leads to inflammation causing symptomatic disease (Yano et al. 2019). Estimates suggest that at least 75% of women experience VVC once during their reproductive years (Sobel 2007, Yano et al. 2019). Between 5% and 9% of women suffer from recurrent VVC (RVVC), diagnosed as four or more symptomatic infections per year (Sobel 2007, Yano et al. 2019). Annually, RVVC affects ~138 million women worldwide and it is estimated that 372 million women have RVVC during their lifetime (Denning et al. 2018). In this review, we will focus on VVC and the role of the interactions between *C. albicans*, the host, and bacterial microbiome during commensalism and disease. Taking this knowledge into account, we will discuss how this translates to physiologically relevant infection models and treatment strategies.

## *Candida albicans*: commensalism and pathogenicity

The host can tolerate a low, moderate, or even high burden of *C. albicans* cells in a nonpathogenic state at epithelial barriers, while an imbalance in homeostasis, such as that of the microbiota due to antibiotic treatment, can promote *C. albicans* pathogenicity, subsequent inflammatory immune responses, and disease (Ardizzoni et al. 2021, d’Enfert et al. 2021, Jacobsen 2023).

**Figure 1.**
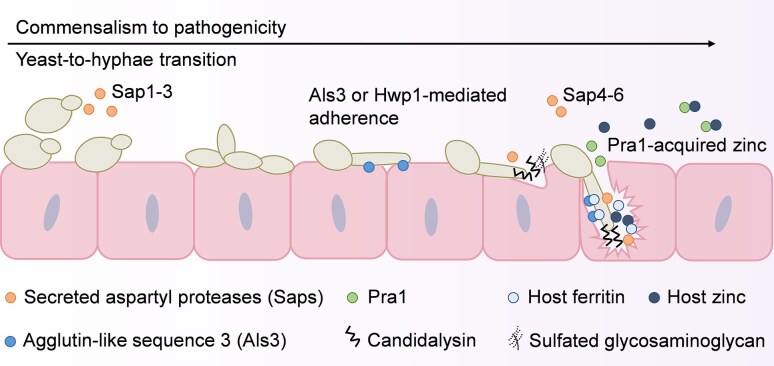
*Candida albicans* factors involved in commensalism and VVC. *Candida albicans* is morphologically diverse and can grow amongst others as yeast, pseudohyphae, or hyphae. The yeast-to-hyphal transition is associated with invasion and can be triggered by various host factors such as 37°C, serum, contact to surfaces, and *N*-acetylglucosamine. Hyphae adhere strongly to the vaginal epithelium *via* the adhesins agglutin-like sequence 3 (Als3) and hyphal wall protein 1 (Hwp1) and can, to some extent, invade the host tissue without causing damage. Once hyphae elongate and invade the epithelium more extensively, secreted candidalysin and secreted aspartyl proteases (Saps) become concentrated within the invasion pocket leading to host damage. However, candialysin can bind to sulfated glycosaminoglycans (GAGs) on the surface of epithelial cells and addition of exogenous sulfated GAGs or the analogue dextran sulfate can protect cells against candidalysin-induced damage. Ferritin and zinc are released from damaged vaginal epithelial cells that can be bound by Als3 and Pra1 to provide the fungus with iron and zinc.

The pathogenicity mechanisms of *C. albicans* have been well investigated but relatively little is known about the commensal stage (Kumamoto et al. 2020). It is hypothesized that by asymptomatically colonizing the human body, this fungus has adapted to its host in a way that allows both a commensal and pathogenic lifestyle (Siscar-Lewin et al. 2022). Host niches can serve as a “commensal virulence school” that enables *C. albicans* to acquire and maintain attributes that support commensal fitness, but also pathogenicity depending on the susceptibility of the host (Hube 2009). For example, the metabolic flexibility of *C. albicans* contributes to its fitness as both a commensal and pathogen (Mayer et al. 2013, Brown et al. 2014a, 2014b). Furthermore, *C. albicans* is a polymorphic fungus that can grow in various morphologies and phenotypes, including pseudohyphae and true hyphae (Anderson and Soll 1987, Gow et al. 2002, Sudbery et al. 2004, Staib and Morschhauser 2007, Pande et al. 2013, Tao et al. 2014), which are important in specific niches or processes. For example, the ability to transition between a yeast and hyphal morphology is central to commensalism and pathogenicity (Naglik et al. 2017, Kumamoto et al. 2020, Liang et al. 2024) (Fig. 1).

Hypha formation by *C. albicans* is induced by traits that are characteristic of the human body such as 37°C, serum, contact to surfaces, and *N*-acetylglucosamine (d’Enfert et al. 2021, Siscar-Lewin et al. 2022). A combination of *in vitro, ex vivo*, and *in vivo* data show that hyphae are highly adhesive, can invade and damage epithelial cells, contribute to biofilm formation, and mediate escape from immune cells (Ermert et al. 2013, Austermeier et al. 2020, d’Enfert et al. 2021). Before invasion, *C. albicans* hyphae can adhere to the epithelium *via* hyphal specific adhesins such as agglutin-like sequence 3 (Als3) (Hoyer et al. 1998) and the hyphal wall protein 1 (Hwp1) (Staab et al. 1999) (Fig. 1). Once adhered, *C. albicans* can form biofilms on the vaginal mucosa (Harriott et al. 2010). Biofilms can contain antifungal resistant cells that persist in the vaginal niche and therefore act as a reservoir for future infection (McKloud et al. 2021). Invasion into epithelial cells is mediated by two processes: induced endocytosis and active penetration (Phan et al. 2007, Dalle et al. 2010, Sun et al. 2010, Wachtler et al. 2012). Induced endocytosis is mostly studied *in vitro*, where it is shown to be triggered by the binding of Als3 or Ssa1 to E-cadherin or N-cadherin on epithelial and endothelial cells, respectively, and is mediated by clathrin-dependent cytoskeletal remodeling of host cells (Phan et al. 2007, Moreno-Ruiz et al. 2009, Sun et al. 2010, Wachtler et al. 2012). Nevertheless, in murine OPC and disseminated candidiasis models, an Ssa1-deficient mutant of *C. albicans* had reduced virulence (Sun et al. 2010). *In vitro*, induced endocytosis of *C. albicans* is cell type dependent, including oral and vaginal epithelial cells (VECs), but not intestinal cells, and is mediated by hyphae but does not require active fungal growth (Dalle et al. 2010, Wachtler et al. 2012). In contrast, active penetration requires *C. albicans* hypha extension that seems to be associated with the release of secreted aspartyl proteases (Saps) both *in vitro* and *in vivo* (Naglik et al. 2003, Dalle et al. 2010, Bruno et al. 2015, Mogavero et al. 2021) (Fig. 1). The Sap enzyme family is diverse in its function with Sap1–3 being secreted by yeast cells and Sap4–6 secreted by hyphae (Naglik et al. 2003).

Importantly, host cell damage is predominantly mediated by the release of the pore-forming peptide toxin candidalysin as mutants lacking candidalysin invade normally, but are largely unable to damage epithelial cells (Moyes et al. 2016, Mogavero et al. 2021). Before secretion, candidalysin is embedded in a precursor protein, Ece1, which consists of a signal peptide, a candidalysin precursor, and seven non-candidalysin Ece1 peptides (NCEPs) (Muller et al. 2024). The NCEPs prevent intracellular autoaggregation of candidalysin sequences and play a role in intracellular Ece1 folding as well as candidalysin secretion. Moderate levels of candidalysin-mediated epithelial damage can facilitate nutrient acquisition. This includes zinc acquisition through Pra1, a zinc-binding molecule that is secreted by *C. albicans* in response to neutral pH, hyphal formation, and zinc limitation (Sentandreu et al. 1998, Citiulo et al. 2012, Sprague et al. 2024). After zinc acquisition, Pra1 associates with Zrt1, a Pra1 receptor and zinc transporter on the fungal cell, to deliver the sequestered zinc to *C. albicans* (Citiulo et al. 2012) (Fig. 1). In addition, iron is likely acquired during invasion *via* Als3-mediated binding of the epithelial iron storage protein ferritin (Almeida et al. 2008). Since its discovery, candidalysin has emerged as an integral virulence factor during oral epithelial-, inflammatory bowel-, systemic-, and alcohol-associated liver disease, as well as during interactions with macrophages (Kasper et al. 2018, Drummond et al. 2019, Ho et al. 2019, Swidergall et al. 2019, Chu et al. 2020, Blagojevic et al. 2021, Li et al. 2022). It has only recently been shown that hyphae and candidalysin secretion are also beneficial for gut colonization in the presence of high levels of bacteria (Liang et al. 2024). The immune system responds to damage caused by candidalysin by inducing a danger response pathway, which drives the recruitment of phagocytes (Moyes et al. 2010, 2016). This can lead to protective immune responses that mediate fungal clearance or contribute to immune dysfunction as in the case of VVC (Richardson et al. 2018).

## VVC

### Clinical aspects of VVC

Common symptoms of VVC include soreness, itching, burning, and redness (Sobel 2007, Yano et al. 2019). Vaginal discharge is also often reported (Sobel 2007). Symptoms of VVC overlap with other dermatological conditions such as eczema, and VVC is therefore clinically diagnosed based on patient history, presentation, and laboratory findings (Saxon et al. 2020). Importantly, diagnostic tests should only be performed in women with signs and symptoms of VVC to prevent overdiagnosis, since as well as causing VVC, yeasts can asymptomatically colonize the vagina of women (Goldacre et al. 1979, Sobel 2007, Solis-Arias et al. 2014, Saxon et al. 2020, Moreira et al. 2021). One study even reporting colonization rates as high as 60% (Fernandes et al. 2022). Frequencies of detection vary, but colonization by *C. albicans, C. glabrata, C. krusei, C. parapsilosis*, and *C. tropicalis* have been reported (Solis-Arias et al. 2014, Moreira et al. 2021). *Candida albicans*, the species most often found to asymptomatically colonize women, is also the main species responsible for causing disease (Solis-Arias et al. 2014, Farr et al. 2021, Moreira et al. 2021, Fernandes et al. 2022). The presence of pseudohyphal or hyphal morphologies is indicative of a *C. albicans*-caused disease, while the presence of yeasts indicates that the disease is caused by non-*albicans Candida* (NAC) species since these species typically do not undergo the yeast-to-hyphal transition (Pekmezovic et al. 2021a, Neal and Martens 2022). *Candida albicans* is therefore quite unique in its pathology compared to NAC species.

### Predisposing factors of VVC

The causes that shift the normally commensal relationship between *C. albicans* and the host toward the development of VVC are multifactorial (Sobel 2007). Factors and conditions damaging the skin or impairing mucosal integrity, disrupting the vaginal microbiome (e.g. douching, sexual intercourse, and antibiotic use), together with conditions associated with high levels of glucose (e.g. uncontrolled diabetes mellitus) or estrogen (e.g. oral contraceptives, pregnancy, and hormone replacement therapy) have been described to predispose to VVC (Sobel 2007, Guzel et al. 2011). The diverse functions and central role of estrogen during VVC will be discussed throughout this review. Furthermore, women with atopy and allergic diseases or bacterial vaginosis (Neves et al. 2005, Sobel 2007, Sobel and Vempati 2024) tend to be more susceptible to VVC. Some studies have identified antibiotic use, followed by sexual intercourse, as the highest risk factors (Yano et al. 2019). Humid weather, feminine hygiene products, the use of over-the-counter antifungals, noncotton underwear, and a history of childbirth have also been described to play a role in the susceptibility to VVC (Guzel et al. 2011, Yano et al. 2019, Fernandes et al. 2022). Importantly, asymptomatic carriage of *Candida* species is more readily detected in the vaginas of women with previous symptomatic VVC infections and women with a history of VVC are more prone to develop a new infectious episode (Giraldo et al. 2000, Fernandes et al. 2022). However, it should be noted that in many cases the cause of developing disease is unknown (idiopathic) and the number of idiopathic flares is similar between VVC and RVVC patients (Yano et al. 2019). It is therefore unclear why only some women develop RVVC, while others only have a single acute episode.

### Genetic predisposition

Genetic predisposition plays a significant role in RVVC susceptibility, which is characterized by common genetic variations rather than severe genetic deficiencies in genes related to the immune system (Rosentul 2009 et al. 2009, Jaeger et al. 2013). There is a higher frequency of mannose-binding lectin (*MBL*) gene polymorphisms (Liu et al. 2006, Donders et al. 2008) and ABO–Lewis blood group with nonsecretor phenotypes among RVVC patients (Chaim et al. 1997). MBL deficiency is associated with immune overcompensation leading to hypersensitivity, atopy, and autoimmune diseases (Borta et al. 2019). Women with RVVC also more commonly have a C → T substitution in the gene encoding for interleukin (IL)-4 that results in increased *IL4* expression, reduced nitric oxide and MBL, and an impaired anti-*Candida* innate immune response (Babula et al. 2005). Similarly, women with polymorphisms in *IL12* are suspected to be more prone to RVVC as a result of higher IL-12 expression (Isakhani 2022 et al. 2022). Variable number tandem repeats in the inflammasome-associated gene *NLRP3* are linked to increased IL-1β and reduced IL-1 receptor antagonist (Ra) levels in RVVC patients (Jaeger et al. 2016). Increased IL-1β and *NLRP3* expression, as well as cytokine release were linked to a polymorphism in sialic acid-binding immunoglobin-like lectin 15 (*SIGLEC15*), which was more common in RVVC patients (Jaeger et al. 2019). Additionally, evidence suggests that mutations in caspase recruitment domain-containing protein 9 (*CARD9*) (Glocker et al. 2009, Vaezi et al. 2018) and in the β-glucan receptor dectin-1 (Ferwerda et al. 2009) predispose to VVC. Although unclear why, African American women are more likely to develop VVC (Foxman et al. 2000, Sobel 2007).

In most cases, the exact causes responsible for driving the onset of VVC are unknown, but it is clear that changes in the relationship between *C. albicans* and factors in the vaginal environment are integral to the development of VVC. Disease is prevented by maintaining balance at the epithelial barrier within the vaginal niche.

### Epithelial recognition and responses during VVC

*Candida albicans* asymptomatically colonizes the vaginal epithelium of most women (Drell et al. 2013). The vaginal epithelium is therefore the first line of defense against disease, where *C. albicans* colonization has to be distinguished from *C. albicans* pathogenicity to either tolerate asymptomatic colonization by the fungus or mount a response to counteract symptomatic infection (Naglik et al. 2011, d’Enfert et al. 2021, Mills et al. 2024). During vaginal colonization and disease, mixed populations of both yeast and filamentous morphologies are found in women (Roselletti et al. 2017, 2019a). Both *in vitro* and *in vivo* findings support that VECs, like other epithelial cell types, discern between asymptomatic colonization and symptomatic infection by monitoring hyphal-associated damage *via* mitogen-activated protein kinase (MAPK) signaling (Moyes et al. 2011, Roselletti et al. 2019a).

*Candida albicans* yeast cells are initially recognized by their cell wall constituents followed by activation of the nuclear factor kappa B (NF-κB) pathway (Moyes et al. 2011). The *C. albicans* cell wall contains various pathogen-associated molecular patterns, including mannan and β-glucan, that can be recognized by pathogen recognition receptors (PRRs) on epithelial cells (Naglik et al. 2011, d’Enfert et al. 2021). Using a Δ*efg1*/Δ*cph1* yeast-locked mutant of *C. albicans*, it was observed that the presence of yeast cells led to lower proinflammatory cytokine release by VECs (Moyes et al. 2011). Supporting this idea is the fact that in women moderate immune activation was observed when *C. albicans* was present in its yeast form (Roselletti et al. 2019a). Additionally, *in vitro*, a mitochondrial-driven type I interferon response increases vaginal epithelium resistance to *C. albicans*-induced damage and prevents activation of recruited neutrophils (Pekmezovic et al. 2021a).

In addition to the hyphal triggers discussed before (see *C. albicans*: commensalism and pathogenicity), estrogen is a specific inducer of hyphal formation (d’Enfert et al. 2021). However, women can asymptomatically be colonized with pseudohyphae and hyphae and the mere presence of these morphologies do not necessarily cause severe epithelial damage, inflammation, or symptoms (Roselletti et al. 2019a). In *in vitro* models with VECs, *C. albicans* hyphae, and an increased fungal burden together with increased levels of host cell damage induce MAPK signaling through MAPK phosphatase-1 (MKP1) and c-Fos activation (Moyes et al. 2011). Similarly, in women the hyphal morphology has been linked to c-Fos activation (Roselletti et al. 2019a). Such late responses are primarily driven by candidalysin-induced host cell damage, which can be prevented by the addition of exogenous sulfated glycosaminoglycans that bind candidalysin (Richardson et al. 2018, Lin et al. 2024). As mentioned before, this danger response pathway triggers cytokine release and immune cell recruitment, which is crucial for the host to remain tolerant to asymptomatic colonization but responsive to symptomatic infection.

### The misdirected immune response during VVC

Depending on the host niche, *C. albicans* can be classified as different pathogen types within the damage response framework (DRF) (Casadevall and Pirofski 1999, Fidel et al. 2020). The DRF is a classification system that determines the outcome of the presence of a microbe based on the immune response (Casadevall and Pirofski 1999) and different classification of *C. albicans* within this framework exemplifies the diverse nature of *C. albicans* (Fidel et al. 2020). For example, in the case of OPC, *C. albicans* is a pathogen that only causes epithelial damage when the immune system is compromised, whereas VVC occurs in otherwise healthy, non-immunocompromised, women and is characterized by strong immune responses (Fidel et al. 2020). While OPC is used as a hallmark to diagnose human immunodeficiency virus (HIV) disease (Samaranayake 1992), the role of T helper (Th) responses during VVC is disputed and the incidence of VVC was reported to be similar in HIV-negative and -positive women (Pietrella et al. 2011, Yano et al. 2012, Apalata et al. 2014, Ge et al. 2022). Yet, T cell responses mediated by vaccination play a role in the protection against RVVC (Edwards et al. 2018). More research is needed to determine the role of adaptive immunity at the vaginal mucosa. During VVC, however, fungal pathogenicity mechanisms are suspected to catalyze an acute innate immune response, which becomes uncontrolled after recruited inflammatory cells fail to clear the disease and inflict collateral tissue damage (Fidel et al. 2004, Yano et al. 2018, Ardizzoni et al. 2021, Cheng et al. 2024). When the host mounts inflammatory responses, the recruited neutrophils deploy neutrophil extracellular traps (NETs) (Urban et al. 2006). Upon NETosis and neutrophil degranulation, high concentrations of neutrophil effector molecules are released such as proteases, myeloperoxidase, and reactive oxygen species (ROS) that, in addition to the *C. albicans*-induced damage, can cause mucosal tissue damage (Wilgus et al. 2013, Hopke et al. 2020). This exacerbates the release of damage-associated molecular patterns (DAMPs), further promoting inflammation and neutrophil recruitment. Thus, VVC immunopathology results from the influx of large numbers of activated neutrophils into the vaginal mucosa, which is maintained by a positive feedback loop that is driven by tissue damage and the release of DAMPs.

### *Candida albicans* and host factors driving immunopathology during VVC

**Figure 2.**
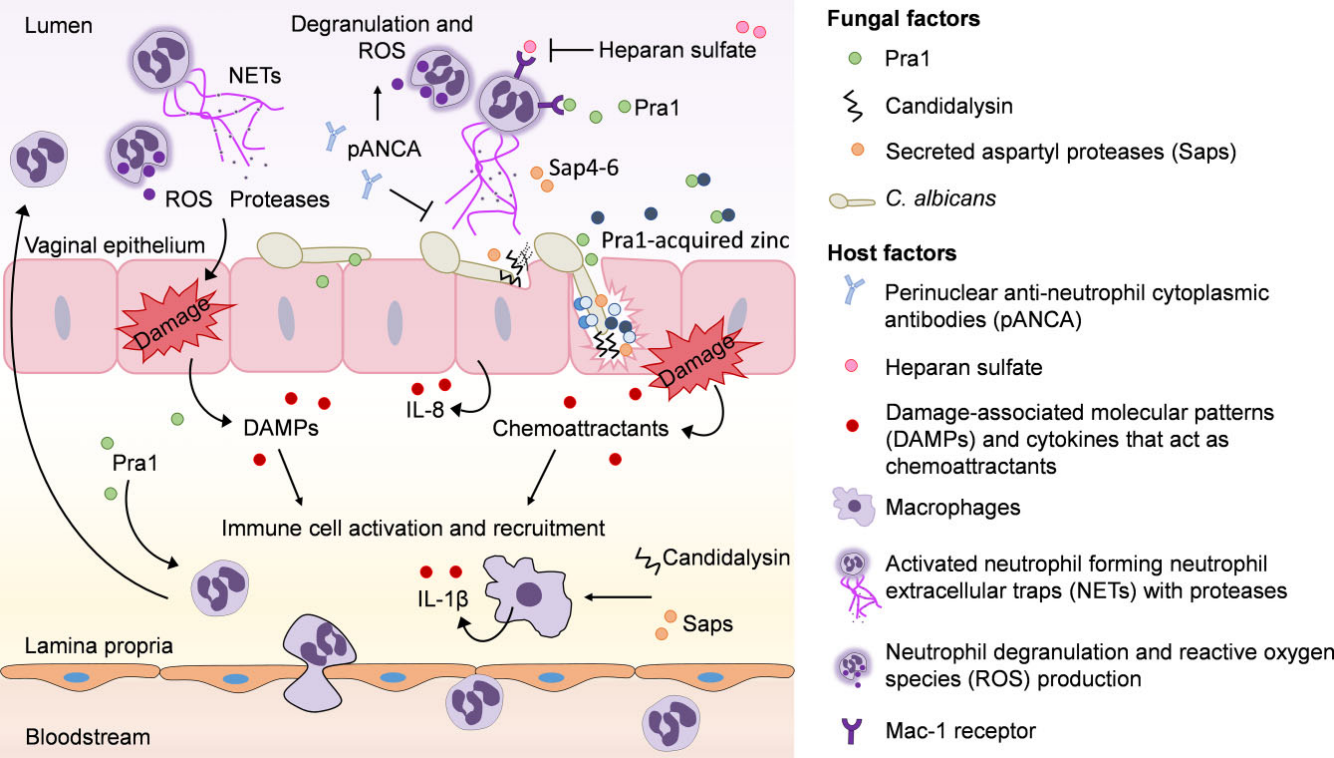
Host response to *C. albicans* factors that trigger inflammation and immunopathology during VVC. Invading *C. albicans* hyphae cause vaginal epithelial damage *via* candidalysin secretion that is concentrated in the invasion pocket, but can also bind to sulfated glycosaminoglycans on the surface of epithelial cells. Candidalysin-mediated damage results in the release of DAMPs and cytokines that trigger an immune response. *Candida albicans* can also directly or indirectly activate immune responses *via* its various virulence factors such as the zinc-scavenging molecule Pra1 and secreted aspartyl proteases (Saps). NLRP3 inflammasome activation of macrophages results in IL-1β release that further promotes the inflammatory state during VVC. Neutrophils are recruited to the vaginal epithelium to control the infection, however, their efficiency to kill *C. albicans* is debilitated by factors present in the vaginal niche such as perinuclear anti-neutrophil cytoplasmic antibodies (pANCA) and heparan sulfate. NET formation, ROS production, and protease release by activated neutrophils exacerbate inflammation.

Several fungal factors have been described to catalyze the onset of VVC immunopathology (Ardizzoni et al. 2021) (Fig. 2). Particularly, hyphae-associated virulence factors drive vaginal mucosal damage that result in the release of DAMPs and proinflammatory cytokines (Peters et al. 2014, Richardson et al. 2018, Roselletti et al. 2019a). Whether a hyperinflammatory response is induced depends on the *C. albicans* strain and its specific yeast or hyphal morphology (Shankar et al. 2020). The fungal zincophore Pra1 serves as a neutrophil chemoattractant and thereby drives hyperinflammation (Soloviev et al. 2007, Roselletti et al. 2023). Candidalysin and Saps further drive neutrophil influx and activation through causing tissue damage that leads to inflammatory cytokine release and activating the NLRP3 inflammasome (Pietrella et al. 2013, Pericolini et al. 2015, Gabrielli et al. 2016, Roselletti et al. 2017, Kasper et al. 2018, Richardson et al. 2018). NLRP3 inflammasome activation contributes to the inflammatory environment *via* IL-1β release (Bruno et al. 2015, Roselletti et al. 2017). In response to hyphae, neutrophils degranulate and induce NETosis in an attempt to facilitate fungal killing (Urban et al. 2006, Hopke et al. 2020). Candidalysin was recently identified as a potent inducer of NETosis (Unger et al. 2023). In addition to contributing to the hyperinflammatory environment, recruited neutrophils during VVC, unlike during oral and systemic candidiasis, are dysfunctional and do not restrict disease by efficiently clearing the fungal burden (Yano et al. 2018, Fidel et al. 2020).

In mice, epithelial cells secrete S100A8/9 alarmins in response to *C. albicans* infection, which lead to robust neutrophil recruitment (Yano et al. 2010). However, heparan sulfate impaired neutrophil function by binding the Mac-1 receptor on neutrophils (Yano et al. 2017) (Fig. 2). NET formation and *C. albicans* killing is primarily mediated by Mac-1 recognition of Pra1 on *C. albicans* hyphae (Yano et al. 2018, Yano and Fidel 2024). Blocking the Mac-1 receptor is therefore hypothesized to prevent clearance of the fungal burden during disease. However, as stated above, Pra1 was found to be a key player in driving VVC pathogenesis in women (Roselletti et al. 2023). In a natural setting, neutrophils are likely exposed to concentrations of Pra1 over a chemotactic gradient that allows neutrophils to migrate and respond. Under zinc limitation, Pra1 will continuously be secreted by *C. albicans* to acquire zinc and grow, which would increase neutrophil influx and inflammation (Citiulo et al. 2012, Roselletti et al. 2023). Through the release of calprotectin by neutrophils, zinc can be further restricted maintaining this fungal-effector-inflammatory loop (Besold et al. 2018). Depending on the location within the vaginal niche, as well as the time during disease, different fungal and host factors may mediate immunopathology. Elevated levels of perinuclear anti-neutrophil cytoplasmic antibodies (pANCA) reported in VVC patients also contribute to VVC immunopathology by prematurely activating ROS production by neutrophils thereby limiting their ability to deploy these mechanisms when encountering the fungus (Ardizzoni et al. 2020). In addition to stimulating hyphal formation, estrogen can affect the host immune response to *C. albicans* to promote disease. Estrogen enables *C. albicans* immune evasion by promoting binding of Factor H on the fungal cell surface, thereby preventing opsonization and phagocytosis (Kumwenda et al. 2022). In addition to fungal and host factors, the vaginal environment is shaped by the microbiota that can have further adverse effects on *C. albicans*.

## The role of the microbiota in VVC

The onset of VVC in some cases is associated with the use of antibiotics, some studies even describe it as the highest risk to develop VVC (Yano et al. 2019). Bacteria of the healthy vaginal microbiota are therefore believed to be essential in maintaining homeostasis by antagonizing *C. albicans* pathogenicity. Compared to the intestinal microbiota, the healthy vaginal microbiota of reproductive-aged women has a low microbial diversity that is dominated by *Lactobacillus* species (Ravel et al. 2011, d’Enfert et al. 2021). The dominating *Lactobacillus* species of the healthy vaginal microbiota may differ depending on geography. Five community state types (CSTs) have been described: CST-I (*L. crispatus*-dominated); CST-II (*L. gasseri*-dominated); CST-III (*L. iners*-dominated); CST-V (*L. jensenii*-dominated); and CST-IV (diverse anaerobes, associated with bacterial vaginosis) (Ravel et al. 2011). Interestingly, a *L. crispatus*-dominated microbiota i.e. CST-I was found to be associated with *C. albicans* colonization (Brown et al. 2019), supporting the idea that *C. albicans* can form part of the healthy vaginal microbiome.

The vaginal bacterial microbiota is highly dynamic especially depending on hormonal changes and CST states can change during menses, pregnancy, or menopause (d’Enfert et al. 2021). Estrogen not only affects the host immune response and *C. albicans* morphology as mentioned before but it also affects the epithelial integrity (Luthje et al. 2013) and lactobacilli population. Estrogen promotes glycogen deposition in the vaginal epithelium and when epithelial cells are lysed or exfoliated, glycogen becomes available in the vaginal niche (Amabebe and Anumba 2018). Glycogen is degraded by host and bacterial amylases into simple sugars that foster the growth of *Lactobacillus* species (Spear et al. 2014, Miller et al. 2016, Nunn et al. 2020). At least in part due to the different optimal pH at which glycogen-degrading enzymes function, glycogen availability and pH were found to influence bacterial growth in a vaginal fluid simulative medium (Jenkins et al. 2023, Navarro et al. 2023). Glycogen metabolism was also shown to be important for *C. albicans* fitness in a murine VVC model (Miao et al. 2023). The dynamic nature of the vaginal microbiota highlights the difficulty of linking specific microbiota changes with the onset of VVC. Indeed, women with VVC show diverse microbiome patterns and not a specific microbiome signature characteristic of VVC has been identified (Liu et al. 2013). Further illustrating the complexity of VVC is the fact that estrogen is a major predisposing factor, while simultaneously promoting *Lactobacillus* colonization. Through their lactic acid production (which also has other diverse roles that are discussed below), these bacteria are generally believed to help maintain a healthy vaginal pH between 4 and 4.5, where *C. albicans* hyphae production is limited (Saporito-Irwin et al. 1995, Boskey et al. 2001, Sobel 2007, Kohler et al. 2012).

Although a *Lactobacillus*-dominated vaginal microbiota is associated with health, recent findings are challenging the role of lactobacilli. In women, antibiotic treatment for bacterial vaginosis was shown to increase the presence of vaginal fungi, predominantly *C. albicans*, and this correlated with an expansion in the lactobacilli population (Armstrong et al. 2024). Furthermore, the overall abundance of *Lactobacillus* species remains comparable between healthy women and women with VVC (Ceccarani et al. 2019, Zhao et al. 2023). A shift in the *Lactobacillus* species present during VVC, i.e. from a *L. crispatus*-dominated to *L. iners*-dominated microbiota with an increase in *Lactobacillus* species diversity and *L. gasseri* abundance, has been described (Ceccarani et al. 2019). While still producing lactate that keeps the vaginal pH low, the type of lactic acid produced may be crucial in determining the capacity of the microbiota to antagonize fungal pathogenicity. *Lactobacillus crispatus* is typically associated with vaginal health in part due to d-lactic acid production that is more antimicrobial than l-lactic acid (Verstraelen et al. 2009, Ravel et al. 2011, Amabebe and Anumba 2018), while *L. iners*, which is often associated with vaginal disease, produces l-lactic acid (Verstraelen et al. 2009, Amabebe and Anumba 2018). Micro-niches may also exist in the vaginal tissue where communities of *Lactobacillus* species are not in close enough range to fully antagonize *C. albicans via* the combined effect of lactate and other mechanisms. Collectively, this may explain why vaginal pH and lactate levels are not greatly altered during VVC (Ceccarani et al. 2019, Zhao et al. 2023). Furthermore, the fact that filamentous morphologies are found in the acidic vaginal environment (Sobel 2007, Roselletti et al. 2019a, 2019b), and that elevated pH levels have been reported in healthy women with *Lactobacillus* species-dominant populations (Ravel et al. 2011), suggest that other antagonizing factors are crucial.

**Figure 3.**
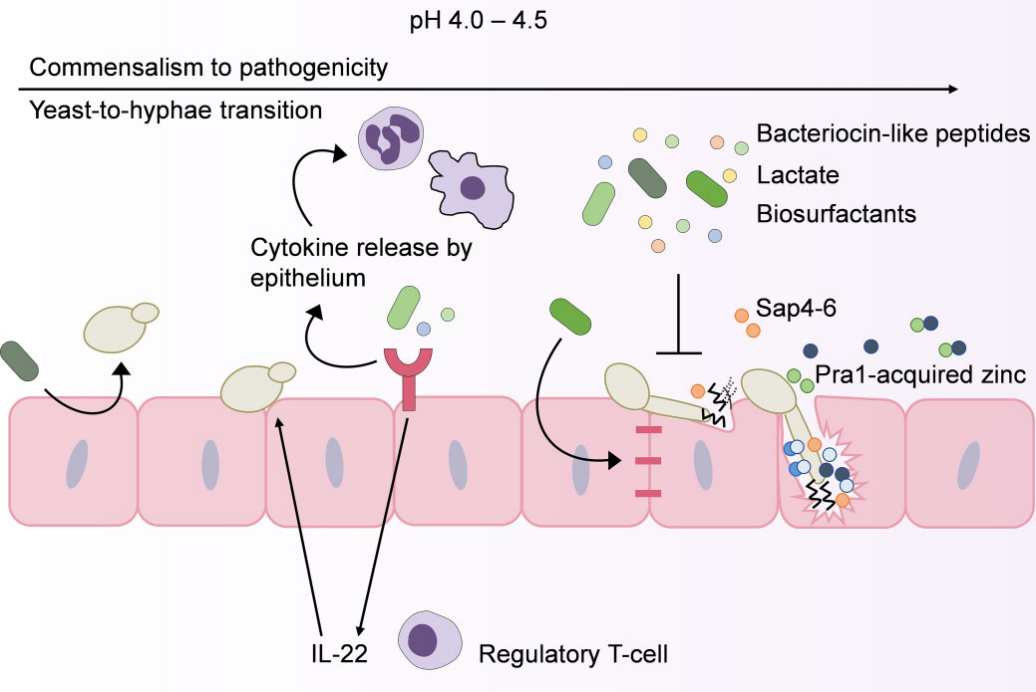
The antagonistic mechanisms of lactobacilli, the predominant members of the vaginal microbiota. *Lactobacillus* species produce several metabolites, including peptides, lactate, and biosurfactants. The production of lactate helps to keep a healthy acidic vaginal pH between 4 and 4.5. Either directly or *via* their secreted metabolites, lactobacilli can prevent *C. albicans* hyphal formation and compete for adhesion to the vaginal epithelium. Lactobacilli can also improve barrier integrity and regulate the immune response at the vaginal epithelium. In addition, lactobacilli can provide IL-22-mediated colonization resistance to *C. albicans*.

A variety of mechanisms have been proposed by which *Lactobacillus* species antagonize *C. albicans* pathogenicity and control asymptomatic colonization or the onset of VVC (Ardizzoni et al. 2021, d’Enfert et al. 2021, Sun et al. 2023, Pedro and Mira 2024) (Fig. 3). Some *Lactobacillus* species and strains can negatively impact *C. albicans* growth, filamentation, and biofilm formation in a contact-independent manner (Strus et al. 2005, Kohler et al. 2012, Poon and Hui 2023, Takano et al. 2023). In addition to lactic acid production as mentioned above, lactobacilli were reported to produce hydrogen peroxide, bacteriocin-like peptides, and biosurfactants that antagonize *C. albicans* (Strus et al. 2005, Hutt et al. 2016, Zangl et al. 2019). Consequently, lactobacilli have been intensively explored as probiotics.

Probiotics are defined as live microorganisms that offer health benefits if taken in adequate amounts (Hill et al. 2014). *Lacticaseibacillus* (formerly *Lactobacillus*) *rhamnosus* GR-1 is the most studied probiotic strain for improving women’s reproductive health, although it must be noted that much more is known about the *L. rhamnosus* strain GG that is associated with the gut (Segers and Lebeer 2014, Petrova et al. 2021). *Lacticaseibacillus rhamnosus* GG for example has been shown to indirectly antagonize *C. albicans* pathogenicity by competing with the fungus for nutrients and adhesion sites to the host (Mailander-Sanchez et al. 2017). Another probiotic *L. rhamnosus* strain has also been shown to indirectly antagonize *C. albicans* by limiting carbon sources that are favored by the fungus, which forces *C. albicans* to metabolically adapt and subsequently compromises its pathogenicity (Alonso-Roman et al. 2022).

Lactobacilli can further indirectly antagonize *C. albicans* pathogenicity by affecting the host (Fig. 3). Lactic acid leads to the increased expression of tight junction proteins in cervicovaginal cells and thereby improves the barrier integrity (Delgado-Diaz et al. 2022). Lactobacilli, therefore, have the potential to prevent pathogens from breaching the vaginal epithelium and protect against pathogen-induced inflammation (Reid et al. 2011). Surface-active molecules, including lipoteichoic acids, enable lactobacilli to not only adhere to host cells but also bind PRRs on immune cells to modulate immunity (Chee et al. 2020). The immunomodulatory properties of lactobacilli have been widely reported (Lebeer et al. 2010, Wells 2011). Although predominantly studied in the context of the gut, this immunomodulatory capacity makes probiotic bacteria even more promising for managing inflammation during VVC. For example, biosurfactants mediated the ability of lactobacilli to reduce leukocyte influx into the vaginas of *C. albicans*-infected mice (De Gregorio et al. 2019, 2020). Lactic acid and short-chain fatty acids (SCFAs) that are produced by lactobacilli also have immunomodulatory functions (Manoharan et al. 2021, Liu et al. 2023a, Ney et al. 2023). Although SCFAs are believed to be more proinflammatory in the vagina compared to in the gut (Amabebe and Anumba 2020), it is still interesting to consider the role of *Lactobacillus*-secreted metabolites in VVC. Both in the gut and vagina, lactobacilli can switch from utilizing sugar to tryptophan as a carbon source (Zelante et al. 2013). As a consequence of tryptophan utilization, indole-3-aldehyde is produced that binds the aryl hydrocarbon receptor to produce IL-22 that provides colonization resistance to *C. albicans* (Fig. 3). Furthermore, probiotic *L. rhamnosus* strains can induce type I interferon responses, similar to the protective responses induced by *Candida* species on VECs (Pekmezovic et al. 2021a, Si et al. 2022), and dampen NLRP3 inflammasome responses during disease (Wu et al. 2018).

## Treatment strategies for VVC

### Implemented treatment strategies

The management strategies for VVC disease have been well established (Sobel 2007, Sobel and Sobel 2018, Yano et al. 2019, Neal and Martens 2022, Cornely et al. 2025). Treatment mostly entail an oral or topical azole with boric acid as a good alternative (Sobel 2007, Sobel and Sobel 2018, Neal and Martens 2022). In pregnant women, who are at higher risk for VVC, the use of oral fluconazole treatment is restricted since it is associated with increased risk of spontaneous abortion and stillbirth (Molgaard-Nielsen et al. 2016). In recent years, the new antifungals ibrexafungerp and oteseconazole have emerged as promising ways to treat VVC (Lee 2021, Sobel and Nyirjesy 2021, Martens et al. 2022, Sobel et al. 2022, Phillips et al. 2023).

### New and explored treatments

To achieve protective immunity, two vaccine candidates for RVVC are being explored in clinical trials: NDV-3A targeting the hyphal virulence factor Als3 (Edwards et al. 2018) and PEV7 that is based on a recombinant Sap2 protein of *C. albicans* cloned into an influenza virosome (De Bernardis et al. 2012).

Antivirulence therapy is a relatively new concept that was mainly proposed as a strategy to reduce the development of drug resistance (Allen et al. 2014). This type of therapy specifically targets *C. albicans* virulence factors, rather than fungal growth or survival (Vij et al. 2021). For example, a zinc-containing gel was used to successfully treat women with RVVC by downregulating the expression of Pra1, which is normally upregulated in *C. albicans* under zinc starvation conditions (Roselletti et al. 2023). Interestingly, lower levels of zinc in the plasma of women with RVVC was first reported over 30 years ago (Edman et al. 1986). This strengthens the idea to continue investigating biomarkers of VVC since it can aid the identification of disease mechanisms and subsequent novel treatment strategies. An important benefit is that, unlike antifungals, antivirulence treatment does not disturb the healthy mycobiota during treatment.

As part of the healthy vaginal microbiota, some *Lactobacillus* species antagonize *C. albicans* pathogenicity and are therefore good probiotic candidates to promote vaginal health. Probiotics are recommended to be used together with antifungals to prevent the onset of VVC following antibiotic use (Shukla and Sobel 2019). Importantly, lactobacilli as probiotics, including live biotherapeutic products with *L. rhamnosus*, are safe to use by women (Dausset et al. 2018, van de Wijgert and Verwijs 2020). In the context of VVC, *Lactobacillus* probiotics were shown to alleviate VVC symptoms either alone (Vladareanu et al. 2018, Oerlemans et al. 2020, Mandar et al. 2023, Mollazadeh-Narestan et al. 2023) or as an adjuvant, complementing the use of an antifungal (Martinez et al. 2009, Pendharkar et al. 2015). These probiotics work when administered vaginally but even orally when it passes through the intestinal tract and reaches the vagina 7 days later *via* skin contact with the perineum (Liu et al. 2023b). *L. rhamnosus* and *L. acidophilus* combined with lactoferrin after clotrimazole therapy improved symptoms and recurrence (Russo et al. 2019).

It is important to stress that even though probiotics were shown to successfully treat VVC symptoms and recurrence (Xie et al. 2017, Oerlemans et al. 2020, Mandar et al. 2023, Mollazadeh-Narestan et al. 2023), contradicting results exist in the literature showing that probiotics are not effective, and there remains controversy around the use and success of probiotics among experts (Falagas et al. 2006, van de Wijgert and Verwijs 2020). Upon review of studies conducted using probiotics it was found that *Lactobacillus* probiotics showed a better efficacy to prevent and treat bacterial vaginosis rather than VVC (van de Wijgert and Verwijs 2020). However, it is acknowledged that many studies using lactobacilli-containing probiotics are not executed with large sample sizes and have methodological shortcomings such as the lack of proper control groups (Falagas et al. 2006, van de Wijgert and Verwijs 2020). The antagonistic mechanisms of lactobacilli are well-studied and there is no doubt that these bacteria can limit *C. albicans* pathogenicity (d’Enfert et al. 2021). Nevertheless, for numerous reasons, the effects of probiotics are difficult to show *in vivo*. Models that are used to study the effects of lactobacilli *in vitro* are too simplified to fully recapitulate the complexity of VVC in women. Furthermore, it is difficult to determine how many bacterial cells are needed and will encounter *C. albicans in vivo* to exert their activity. Probiotic lactobacilli that elicit an appropriate immune response in the vaginal niche may be crucial to control VVC disease. While clearly showing promise, there is a need to better evaluate *Lactobacillus* probiotics in the context of the vaginal niche to improve their efficacy in preventing and treating VVC. One could argue that we may learn lessons from probiotic lactobacilli on how to keep *C. albicans* pathogenicity in check and base future prevention and treatment strategies for VVC on these principles.

Therapies aimed at modulating the dysregulated inflammatory response during VVC may prove effective in reducing disease severity and alleviating symptoms. The protective role identified for type I interferons in modulating neutrophil activation and increasing epithelial resistance to disease warrants further investigation to exploit this pathway for immunotherapy in VVC (Pekmezovic et al. 2021a, 2022). Nevertheless, therapeutic type I interferons are not yet available and high costs are associated with interferon-based treatments for other diseases (Nguyen et al. 2019). Anakinra (recombinant IL-1 receptor antagonist) (Cvetkovic and Keating 2002) could aid in breaking the self-propagating IL-1α- and IL-1β-mediated inflammation and canakinumab (an IL-1β neutralizing antibody) (Dhimolea 2010) could be promising to nullify the proinflammatory effects of the cytokine IL-1β during RVVC. Inhibitors of the inflammasome, which is responsible for IL-1β release, could also be explored in the context of RVVC since some compounds have already been shown to be effective in clinical trials for the treatment of inflammatory disease (Zhang et al. 2023). It should be noted that immunotherapy needs more specialized intervention that may not be readily available in all clinical settings worldwide and can be too expensive to be accessed by all individuals. Nevertheless, if efficacious, short-term immunotherapy may prove more cost effective and safe than long-term antifungal maintenance therapy.

While there is still much unknown about how *C. albicans* catalyzes the onset of immunopathology during VVC, several virulence factors of *C. albicans* have been well characterized. This allows us to exploit this knowledge to directly antagonize its pathogenicity during disease. It has been suggested to block the *C. albicans*-secreted peptide toxin candidalysin and its downstream effects on the immune response (Bruno et al. 2015, Richardson et al. 2018). In a VVC mouse model, the addition of dextran sulfate, shown to bind candidalysin, was sufficient to reduce vaginal tissue damage and inflammation (Lin et al. 2024). Nanobodies that bind and neutralize candidalysin can reduce vaginal epithelium damage, cytokine release, and subsequent neutrophil activation (Valentine et al. 2024). Thus, suggesting a potential for anti-candidalysin nanobodies to alleviate inflammation in women with VVC. The neutralization of secreted candidalysin would help to dampen immune activation and inflammation, halting the hyperinflammation at its onset. The nanobodies can be applied, for example, together with azole treatment (Valentine et al. 2024). Once the fungal burden is reduced *via* fluconazole treatment, candidalysin would be secreted to a lesser extent and inflammation together with symptoms would be reduced. After combined azole and nanobody treatment, maintenance azole therapy can be initiated until the next severe episode, where azole treatment can be combined with the nanobodies to control disease and inflammation.

Disease prevention is key but it remains a double-edged sword. On one hand prevention is important since RVVC is extremely difficult to treat, while on the other hand it is difficult to prevent vaginal dysbiosis when it is not always clear which trigger may result in VVC. Additionally, women cannot always avoid certain predisposing factors such as increased estrogen levels during pregnancy (Sobel 2007) and genetic predisposition (Jaeger et al. 2013). In many cases *C. albicans* remains “dormant” as a colonizer until its pathogenicity is triggered resulting in inflammation (Faria-Goncalves et al. 2020, Fernandes et al. 2022). Limiting fungal burden and inflammation thus seem most promising to reduce VVC symptoms. To bring new treatments to market, the development of promising therapy options that have been studied over the last years should be prioritized. Ideally future RVVC treatment would entail personalized treatment strategies based on lifestyle. However, we are far from this being achievable since pathogenesis, especially with regards to recurrence, remains largely unknown.

## Studying vaginal *C. albicans* disease

### *In vitro* models

VVC is studied *in vitro* using the cell lines VK2/E6E7 (healthy vaginal mucosal tissue that was immortalized by retroviral transduction) (Fichorova et al. 1997) and A-431 (stems from a vulval epidermoid carcinoma) (Schaller et al. 2006, Hernandez and Rupp 2009). A reconstituted vaginal epithelium model based on A-431 cells exists that histologically mimics the vaginal mucosa (Schaller et al. 2006). Neutrophils can be integrated into this model either directly in contact with the vaginal epithelium, or indirectly in a cell culture insert to allow for migration toward the infected vaginal epithelium (Schaller et al. 2006, Valentine et al. 2024). Insight into the immune responses to vaginal *C. albicans* disease can be acquired by stimulating isolated primary immune cells with supernatant from *C. albicans*-infected VECs (Pekmezovic et al. 2021a, Valentine et al. 2024). Furthermore, isolated human neutrophils and macrophages can be stimulated with *C. albicans in vitro* to study this fungus’ interaction with immune cells in isolation (Urban et al. 2006, Kasper et al. 2018).

Currently, most studies predominantly use the well-characterized wildtype *C. albicans* strain SC5413 (and/or its derivatives), which is a highly virulent blood isolate (Gillum et al. 1984). It remains unclear to which extent SC5314 represents the characteristics of strains causing VVC. In recent years, unique characteristics of SC5314 have been identified that question its suitability as a representative *C. albicans* strain (Glazier et al. 2023, Lohse et al. 2023, Iracane et al. 2024). In the context of VVC, *C. albicans* isolates have been shown to have divergent effects regarding their interaction with neutrophils and VECs (Shankar et al. 2020, Sala et al. 2023). The *C. albicans* strain SC5314 is also tolerant toward lactic acid (Lourenco et al. 2018), while other *C. albicans* strains have been found to be affected by lactic acid depending on the culture conditions (Zangl et al. 2020). It would therefore be of interest to include more *C. albicans* isolates from asymptomatic women and women with VVC in future studies. Currently to represent the vaginal microbiota in models, VECs can be colonized with *Lactobacillus* species prior to infection with *C. albicans* (Graf et al. 2019, Pekmezovic et al. 2021b, Alonso-Roman et al. 2022). Another study imitated the vaginal metabolome as a proxy for the *Lactobacillus*-dominated microbiota (Zahra et al. 2023). Some studies have included lactobacilli and/or hormones to study their effects on vaginal *C. albicans* infection and found that these can downregulate *C. albicans* pathogenicity and inflammatory cytokine release *in vitro* (Wagner and Johnson 2012, Alves et al. 2014). This illustrates the flexibility of *in vitro* infection models to adapt and increase their physiological relevance depending on the scientific question.

**Figure 4.**
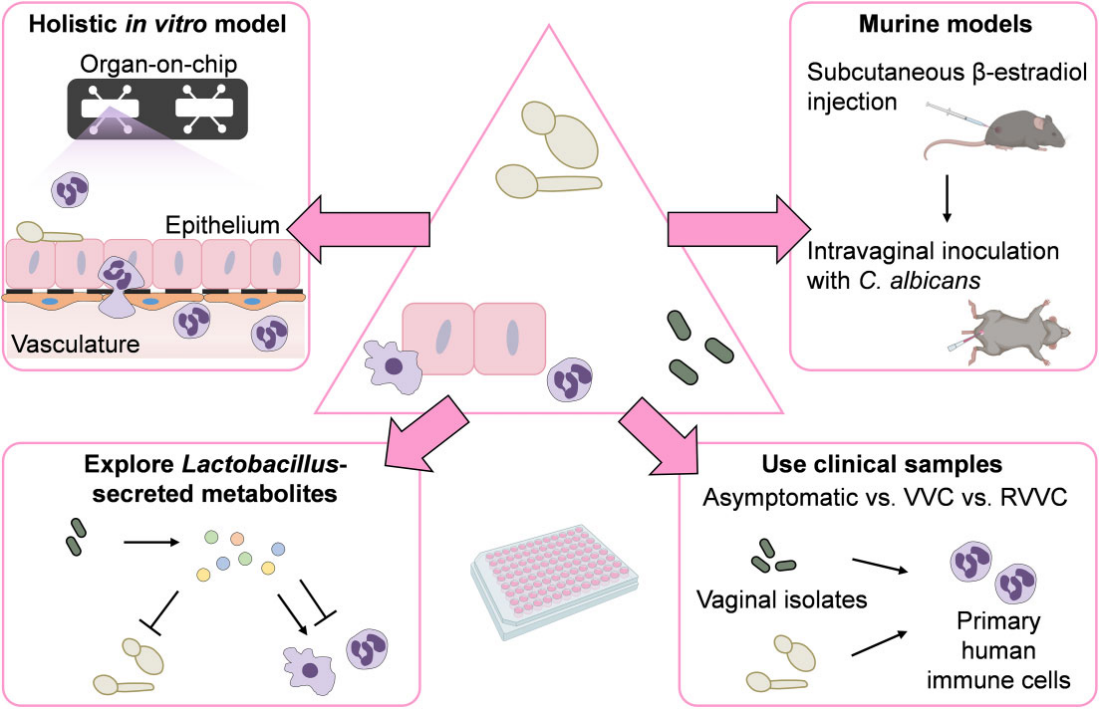
Holistic approach to studying VVC and RVVC. To study the multifactorial disease VVC it is important to use all tools to our disposal. A variety of *in vitro* and *in vivo* models can be employed in concert to study *C. albicans* pathogenicity and the immune response during VVC. Murine models and more physiologically relevant, complex organ-on-chip models can be used to investigate immunity and treatment strategies. Other *in vitro* models can employ samples from women that are asymptomatically colonized with *C. albicans* or with VVC to investigate the pathogenicity of *C. albicans* isolates and disease pathology. Furthermore, the use of probiotic *Lactobacillus* species and their secreted metabolites can be explored using *in vitro* infection models. Created in part with BioRender.com.

Over the past decades, developments in microfluidics, cell culture, and bioengineering led to the introduction of organ-on-chip models that bridge the gap between minimalistic *in vitro* and *in vivo* infection models to offer a more physiologically complex system (Alonso-Roman et al. 2024). An organ-on-chip can be defined as a microfluidic setup, where tissue is cultured to mimic specific aspects of human physiology (Alonso-Roman et al. 2024). Several organ-on-chip models have been developed to mimic various aspects of the female reproductive system (Xiao et al. 2017, Alonso-Roman et al. 2024, Silva et al. 2024). Recently, a vagina-on-chip model included microbiota members associated with a healthy or dysbiotic vaginal microbiota (Mahajan et al. 2022). Compared to standard well plate infection models, organ-on-chips can facilitate long-term studies since media perfusion allows nutrient replenishment and waste product removal. This is especially useful when colonizing the mucosa with bacteria that are typically fast growing and where overgrowth can be flushed out to maintain a stable microbial community. Unfortunately, few of these models have incorporated *C. albicans* (Alonso-Roman et al. 2024). Ideally, organ-on-chip models should include cells isolated from women that are healthy or have VVC that can be infected with *C. albicans* isolates originating from women (Fig. 4). This will not only allow to study the interaction between the host and *C. albicans* during VVC but could form the basis for evaluating and developing personalized treatment strategies. Future VVC-on-chip models would include vaginal microbiota, where dysbiosis can be induced by using, for example, antibiotics to mimic VVC predisposition *in vitro* and/or include immune cells to model the immunopathogenesis of VVC. Furthermore, it would be integral to include estrogen to increase physiological relevance.

One main shortcoming of current models is that the commensal-to-pathogen switch of *C. albicans* cannot be modeled. During commensalism in women, several factors, including the healthy microbiota, reduce the expression of *C. albicans* virulence traits such as hyphae formation (d’Enfert et al. 2021). However, in *in vitro* infection models where *C. albicans* is cultured with host cells, pathogenicity cannot be suppressed and host damage cannot be limited. The addition of a predisposing factor, e.g. antibiotic use, can therefore not result in disease. Another shortcoming of current *in vitro* models is that neutrophil dysfunctionality during VVC can only be modeled using standard infection models that are supplemented with previously identified dysfunctionality factors like heparan sulfate (Yano et al. 2017) or pANCAs (Ardizzoni et al. 2020). Consequently, *in vitro* infection models cannot be used to identify such factors. Dysfunctionality factors would normally co-occur in the vaginal niche, it would therefore be important to perform future experiments in a setting with the vaginal epithelium and immune cells where factors responsible for neutrophil dysfunctionality are combined. Similarly, serum can be used to include active immune elements such as those of the complement system to better represent the physiological conditions during VVC.

### *In vivo* studies

VVC can be studied more comprehensively *in vivo*. While ethically challenging, a previous study confronted women intravaginally with *C. albicans* to study the development of disease in real time (Fidel et al. 2004). This study was crucial in confirming that symptomatic disease is caused by the extent of inflammation that is driven by the innate immune response. In murine *in vivo* models, the vagina is not naturally colonized by *C. albicans* and estrogen needs to be administered to allow infection with the fungus, which is otherwise cleared (Cassone and Sobel 2016). Although known murine models do not completely recapitulate VVC in women, these models do provide important insights into the complex interactions between the fungus and host. In addition to *C. albicans* not being a natural commensal of the murine vagina (in standard laboratory models), there are differences in immunity and vaginal pH (murine models tend to be more neutral), as well as the fact that the microbiota is not dominated by lactobacilli (Cassone and Sobel 2016). Differences between vaginal disease in mice and women have been described (Roselletti et al. 2019b). The associated morphology of *C. albicans* during disease in women is yeast and pseudohyphae, while true hyphae are found in mice. Furthermore, differences in the expression of *C. albicans* virulence genes, specifically *SAP2* and *ECE1*, were observed with both being equally expressed in women and *ECE1* being more dominantly expressed in mice. Nevertheless, findings obtained from *in vitro* and *in vivo* models together with patient samples all indicate that Saps and candidalysin can play a role during disease (Schaller et al. 2003, Pericolini et al. 2015, Roselletti et al. 2017, Richardson et al. 2018). This exemplifies how different infection models can be used to study the mechanisms of VVC disease and that these findings can be validated in patients. With data in women being limited it is important to study VVC by combining the different models at our disposal (Fig. 4). Nevertheless, to decrease the number of animals used during studies, the physiological relevance of current *in vitro* models should be improved. For example, we have recently established an A-431 epithelial model in acidified culture media (Roselletti et al. 2023).

To validate *in vitro* experimental results and to identify factors that play a role during disease, comprehensive VVC cohorts are needed. Data should be collected regularly from women over at least a 1-year period to give clarity on the onset and development of symptomatic infection. Sampling would allow the gathering of information regarding the contents of vaginal flushes and swabs, including the vaginal microbiota (especially lactobacilli populations), pH, as well as *C. albicans* isolates and its morphology. Collected samples should be stored in case of future analysis involving metabolomics and cytokine measurements. In addition, levels of known factors involved in immunopathology, such as heparan sulfate and Pra1 (Yano et al. 2017, Roselletti et al. 2023), could be measured over time to see if they fluctuate and to determine if specific changes are indicative of VVC onset and development.

While several *C. albicans* factors are known to play a role during VVC, the degree to which these fungal factors induce immunopathology may be highly dependent on the *C. albicans* isolate causing the disease. On the other hand, host factors will be determined by behavioral practices and the genetic background of women and will fluctuate throughout a women’s life. Specific combinations of fungal and host factors therefore likely contribute to varying degrees to VVC pathogenesis during different stages of disease. Well-conducted cohort studies could help to stratify patients into subsets depending on the fungal and host factors exacerbating disease and this information could be used in future to personalize women’s treatment accordingly.

In addition to monitoring fungal disease markers, monitoring shifts in vaginal metabolites could lead to the discovery of metabolic biomarkers for the onset of VVC or indicators of disease reoccurrence. Detection of such biomarkers could help to guide specific treatment regimens. Questionnaires regarding lifestyle routines and changes would allow a better patient stratification and to link specific patient groups to findings in the lab.

To accurately determine the impact of probiotics on VVC, clinical trials should include more patients with more comprehensive control groups and correct for self-treatment (Falagas et al. 2006, van de Wijgert and Verwijs 2020). Many studies lack placebo groups and are not blinded, which introduces bias and leads to results that cannot be objectively used to assess the efficacy of lactobacilli-containing probiotics. While the resolution of symptoms, the presence of *C. albicans*, and recurrence of disease are important measures to quantify the efficacy of probiotic use, it may be useful to include comprehensive profiling of disease markers and inflammatory cytokines to better characterize the effect of probiotics on the host. In line with this, in-depth characterization of the immunomodulatory properties of probiotic *Lactobacillus* species using immune cells from uncolonized women, women asymptomatically colonized by *C. albicans*, women with VVC, and RVVC is needed (Fig. 4). Such a study can be extended by conducting similar experiments using *Lactobacillus* species that naturally colonize the vaginal microbiota to gain insight into their immunomodulatory properties *in vivo* and role in VVC. Especially since the role of lactobacilli in VVC susceptibility is not as well characterized as in the intestinal environment. *Lactobacillus* species-derived metabolites can also be further investigated for their immunomodulatory and antagonistic activities. By identifying metabolites that can augment the probiotic efficacy of lactobacilli, we can expand available treatment options for VVC (Fig. 4).

## Conclusions

VVC is a multifactorial disease, where the combined effects of host, microbial, and pathogen-related factors contribute to disease development. The combination of factors leading to disease likely differs between women and specific patient subsets can be identified with unique patterns of disease onset with regards to the catalyzing fungal, host, and microbiota factors. In this review, we focused on *C. albicans* VVC and not VVC caused by NAC species, which further highlights the need for patient stratification. The complicated nature of VVC requires that fungal, host, and microbial factors are studied concomitantly. Nevertheless, to gain mechanistic insight it is necessary to delineate the role of specific factors during VVC. It is therefore necessary to exploit all currently available infection models alongside one another to study VVC and to improve their translational capacity. This will allow us to improve our understanding of VVC pathology, to optimize treatment strategies in cases where maintenance azole therapy is not feasible, and to minimize the development of antifungal resistance. By exploring alternative treatment strategies, we expand on VVC treatment options that can be developed into therapeutic approaches in the future and made available to women as part of personalized medicine.
